# Identifying individuals with recent COVID-19 through voice classification using deep learning

**DOI:** 10.1038/s41598-021-98742-x

**Published:** 2021-09-27

**Authors:** Pichatorn Suppakitjanusant, Somnuek Sungkanuparph, Thananya Wongsinin, Sirapong Virapongsiri, Nittaya Kasemkosin, Laor Chailurkit, Boonsong Ongphiphadhanakul

**Affiliations:** 1 Chakri Naruebodindra Medical Institute, Faculty of Medicine Ramathibodi Hospital, Mahidol University, Bangkok, Samut Prakan Thailand; 2 Department of Communication Sciences and Disorders, Faculty of Medicine Ramathibodi Hospital, Mahidol University, Bangkok, Thailand; 3 Division of Endocrinology and Metabolism, Department of Medicine, Faculty of Medicine Ramathibodi Hospital, Mahidol University, Rama 6th Road, Bangkok, 10400 Thailand

**Keywords:** Biomarkers, Medical research

## Abstract

Recently deep learning has attained a breakthrough in model accuracy for the classification of images due mainly to convolutional neural networks. In the present study, we attempted to investigate the presence of subclinical voice feature alteration in COVID-19 patients after the recent resolution of disease using deep learning. The study was a prospective study of 76 post COVID-19 patients and 40 healthy individuals. The diagnoses of post COVID-19 patients were based on more than the eighth week after onset of symptoms. Voice samples of an ‘ah’ sound, coughing sound and a polysyllabic sentence were collected and preprocessed to log-mel spectrogram. Transfer learning using the VGG19 pre-trained convolutional neural network was performed with all voice samples. The performance of the model using the polysyllabic sentence yielded the highest classification performance of all models. The coughing sound produced the lowest classification performance while the ability of the monosyllabic ‘ah’ sound to predict the recent COVID-19 fell between the other two vocalizations. The model using the polysyllabic sentence achieved 85% accuracy, 89% sensitivity, and 77% specificity. In conclusion, deep learning is able to detect the subtle change in voice features of COVID-19 patients after recent resolution of the disease.

## Introduction

The COVID-19 pandemic has caused enormous health, social and economic burdens. SARS-CoV-2, the virus causing the disease, affects multiple body structures and organs. It infects host cells mainly through binding to ACE2, which has been established as a receptor for the SARS-CoV-2 virus as well as for SARS-CoV-1, enabling the virus to enter host cells. ACE2 is expressed in multiple tissues. The highest expression levels are reported in the small intestine and the lowest in blood vessels and muscle^1^. The respiratory tract is an important site of SARS-CoV-2 infection and disease morbidity. This may be explained by the high expression of ACE2 in human epithelium^2^. Moreover, ACE2 is expressed in oral mucosa^3^ and can also cause loss of smell and taste. Human voice generation is a coordinated function of multiple body structures, including the lungs, vocal folds and laryngeal muscle. About a quarter of patients with mild to moderate COVID-19 have been found to have dysphonia, and interestingly, the expression of ACE2 has also been demonstrated in the vocal folds^4^. Whether there is a subclinical persistence of voice abnormality after recovery from SARS-CoV-2 infection is currently unknown. These signal analyses are an emerging noninvasive voice biomarker for COVID-19 infection.

Recently deep learning has attained a breakthrough in model accuracy for the classification of images due mainly to convolutional neural networks (CNN). Not limited to image classification, CNN has been widely used in converting non-image datasets into 2D or 3D datasets. For voice classification, successful implementations include the classification of singing voice^5^, acoustic scene classification^6^, and audio events classification^7^. In the present study, we hypothesized that subtle voice changes could occur post COVID-19 infection. We attempted to investigate the presence of subclinical voice feature alteration in COVID-19 patients once the disease had resolved, and the ability of artificial intelligence using CNN to classify patients based on past history of COVID-19.

## Materials and methods

### Study sample

This was a prospective study of 76 post COVID-19 patients seen at the outpatient clinic at Chakri Naruebodindra Medical Institutes (CNMI) between May and June 2020. The study was approved by the Faculty of Medicine Ramathibodi Hospital Institutional Review Board. All methods were performed in accordance with the relevant guidelines and regulations. All participants gave their written informed consent before participating in the study. All post COVID-19 patients were more than 8 weeks after onset of symptoms at the time of the study. The exclusion criteria included pregnancy, breastfeeding, uncontrolled hypertension (systolic blood pressure > 160 mmHg or diastolic blood pressure > 100 mmHg), acute myocardial infarction or stroke in past 6 months, history of substance abuse, neurological disorders, current mental health difficulties, active smoking or having stopped smoking for not more than 6 months, alcohol consumption of more than 7 units of alcohol per week, and a history of speech and/or voice disorder such as apraxia of speech, functional articulation disorder, dysarthria, cleft lip/palate, tongue or teeth abnormality, oral occlusion, laryngeal abnormality, or neurological voice disorders. For controls, 40 healthy individuals with no underlying disease were recruited from back-office staff working at CNMI.

### Voice recording

Patients who met the screening criteria were interviewed using a predefined questionnaire to collect demographic data and determine the duration of the disease. Three voice recordings were collected from each participant using a plug-in microphone on a mobile phone. The recordings consisted of a persistent ‘ah’ sound for 5 s, a Thai polysyllabic sentence selected by a voice specialist for vocal apparatus analysis, and a cough sound. The voice recordings were mono-channel and sampled at 44,100 Hz with a maximum duration of 30 s. Both the training and testing set were binary labeled.

### Audio preprocessing and train-test split of the dataset

Each voice sample was divided into 100 ms (ms) subsamples and a log-mel spectrogram was computed using the Python Librosa package. The dimension of each subsample array was 128 × 32. The 2D data array was then converted to 3D suitable for downstream learning by adding a dimension containing identical 2D arrays as the original 2D array. Eighty percent of the total voice records were used as the training set, and the others as the testing set.

### Neural network architecture, training and cross validation

Building and training of the neural network was performed on Tensorflow version 2 (Google, Mountain View, California, USA). We used the VGG19 pre-trained neural network for both pre-train transfer learning and model training. The VGG19 is a widely used CNN, particularly for image classification and computer vision problems due to its in-depth structure and good performance. For transfer and retraining of the VGG19 CNN, the output layer of the VGG19 was dropped and two dense layers of 64, 32 fully connected units, each with batch normalization were added. The new output layer was added with one output unit and a sigmoid activation. A 2D CNN layer was prepended the input of the pretrained VGG19. The input layer of the full transfer learning model was 128 × 32 × 1 in dimension. All layers of the modified VGG19 were made untrainable except for the last five layers to make the pre-trained CNN more suitable for the new voice dataset. Three-fold cross validation was used to assess the performance of the trained neural network. Each fold comprises 78 training samples and 38 training samples. We used a binary cross entropy loss function as our study was a binary classification problem. ADAM optimization was used for the gradient descent with a learning rate of 0.01. Parameters used during training were batch size 32, maximum training epochs 600, percentage of training sample set aside randomly for validation 20% and the matric monitored was area under the curve of the performance of the validation set.

### Shannon entropy calculation

Shannon entropy of each voice type in all subjects was calculated using the Python AntroPy package.

### Statistical analyses

Data were expressed as mean ± SD unless specified otherwise. Multiple logistic regression models were used for assessing potential associated factors. A *p* value less than 0.05 was considered statistically significant. All analyses were performed using Stata Statistical Software, Release 12 (StataCorp, College Station, TX, USA).

## Results

Clinical characteristics of study participants are shown in Table 1. In this sample, patients with COVID-19 were older and had higher BMI than controls. The proportion of males to females was higher in the COVID-19 group than in the control group. Logistic regression analyses with three-fold cross-validation were used to assess the diagnostic values of clinical characteristics to predict recent COVID-19. A model with clinical characteristics including age, sex, and BMI could predict recent COVID-19 with diagnostic values shown in Table 2.

**Table 1:** Clinical characteristics of participants with past COVID-19 and controls (mean ± SE).

Variables	COVID-19 (n = 76)	Non COVID-19 (n = 40)	*p* value
Age (year)	40.26 ± 1.40	30.08 ± 0.96	< 0.001
Female (%)	39 (51.3%)	37 (92.5%)	< 0.001
BMI (kg/m^2^)	24.1 ± 0.5	22.2 ± 0.7	< 0.05

Participants with recent COVID-19 were older, had higher BMI and were more likely to be female, than controls.

**Table 2:** Diagnostic values of clinical characteristics in predicting recent COVID-19.

	Accuracy	Sensitivity	Specificity	PPV	NPV	Kappa
Fold 1	0.77	0.67	1.00	1.00	0.57	0.55
Fold 2	0.67	0.80	0.43	0.71	0.55	0.24
Fold 3	0.72	0.88	0.43	0.73	0.67	0.33
	0.72 ± 0.05	0.78 ± 0.11	0.62 ± 0.33	0.82 ± 0.16	0.59 ± 0.06	0.38 ± 0.16

**Figure 1:**
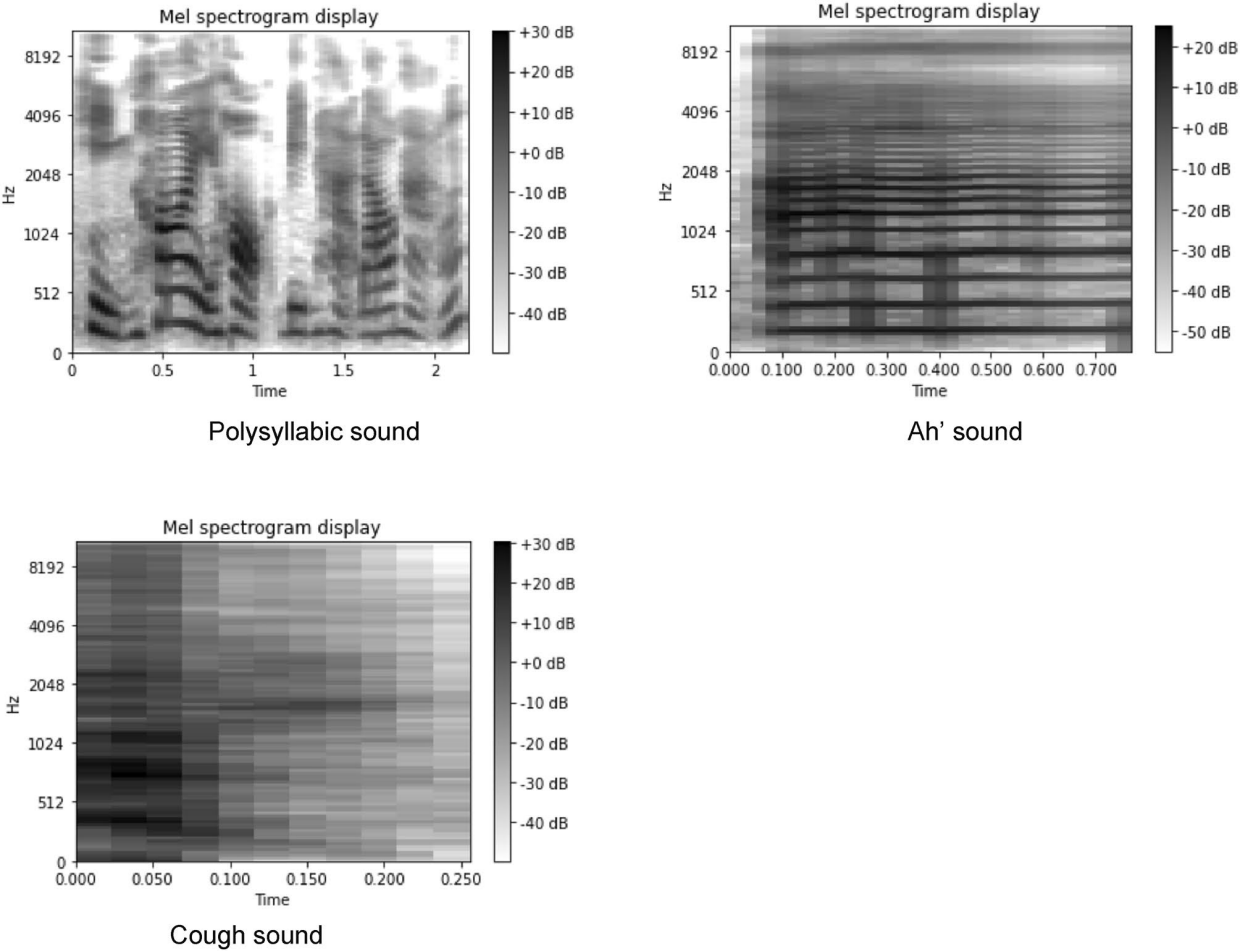
Mel-spectrogram of the 3 voice types from a study subject.

Examples of the mel-spectrogram of the 3 voice types from a study subject were shown in Fig. 1. Table 3 shows the classification performance of CNNs using various voice types. All models were reasonably successful in distinguishing patients with previous COVID-19 from controls. The performance of the model using the polysyllabic sentence yielded the highest classification performance of all models (Table 3A–C). The coughing sound produced the lowest classification performance while the ability of the monosyllabic ‘ah’ to predict the recent COVID-19 was between the other two vocalization types.

**Table 3:** The diagnostic performance of the (A) polysyllabic sentence ‘Hing-Hoy-Hor-Bin-Ha-Dao-Hang’, (B) ‘ah’ sound, (C) cough sound and convolutional neural networks (CNN) to classify recent COVID-19.

	Accuracy	Sensitivity	Specificity	PPV	NPV	Kappa
**A**						
Fold 1	0.77	1.00	0.44	0.72	1.00	0.48
Fold 2	0.82	0.96	0.57	0.80	0.89	0.58
Fold 3	0.85	0.97	0.50	0.85	0.83	0.54
	0.81 ± 0.04	0.98 ± 0.02	0.50 ± 0.07	0.79 ± 0.07	0.91 ± 0.09	0.53 ± 0.05
**B**						
Fold 1	0.74	0.78	0.69	0.78	0.69	0.47
Fold 2	0.82	0.88	0.71	0.85	0.77	0.60
Fold 3	0.77	0.76	0.80	0.92	0.53	0.48
	0.78 ± 0.03	0.81 ± 0.05	0.73 ± 0.05	0.85 ± 0.05	0.66 ± 0.10	0.52 ± 0.26
**C**						
Fold 1	0.71	1.00	0.27	0.68	1.00	0.31
Fold 2	0.56	0.72	0.29	0.64	0.36	0.01
Fold 3	0.73	0.82	0.44	0.82	0.44	0.27
	0.67 ± 0.07	0.85 ± 0.12	0.33 ± 0.08	0.71 ± 0.08	0.60 ± 0.28	0.19 ± 0.13

**Figure 2:**
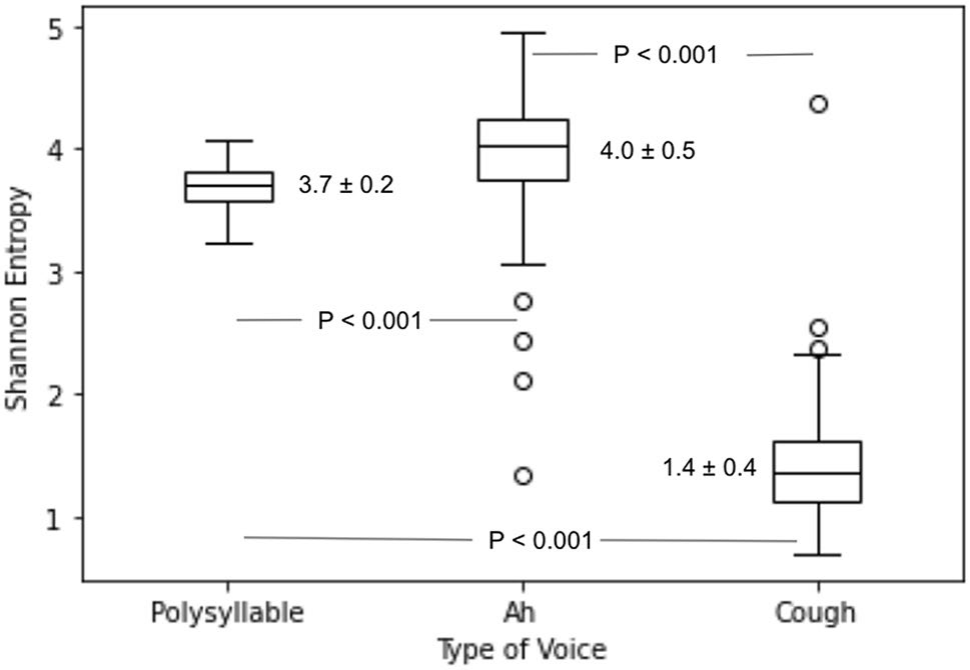
Shannon entropy of the 3 voice types.

We further investigate if the information content of voices as measured by the Shannon entropy may in part be responsible for the better performance of the polysyllable voice. The boxplot of Shannon entropy of each type of voice from all subjects is shown in Fig. 2. The entropy of the polysyllable voice and that of the ‘ah’ voice were significantly higher than that of the cough voice. The entropy of the polysyllable voice was significantly lower than that of the ‘ah’ voice despite that it showed better classification performance than the ‘ah’ voice.

As clinical characteristics of participants with or without recent COVID-19 were not well-matched, we further used multivariate logistic regression analyses to investigate if voice can predict recent COVID-19 independently of age, gender and BMI. Clinical characteristics and the values extracted from the CNN of each fold were shown in Table 4. In most of the datasets in the threefold cross validation, voice characteristics of the polysyllabic sentence as extracted by the CNN were significantly associated with recent COVID-19 independently of age, gender and BMI, as shown in Table 5.

**Table 4:** Clinical characteristics and features extracted from CNN of each fold.

	Sentence	Ah sound	Cough sound
**Fold 1**			
Age (year)		37.1 ± 10.7	
BMI (kg/m^2^)		22.7 ± 4.3	
Female (%)		73.7	
CNN extracted feature	0.82 ± 0.39	0.59 ± 0.49	0.89 ± 0.31
**Fold 2**			
Age (year)		35.7 ± 12.1	
BMI (kg/m^2^)		23.5 ± 4.0	
Female		66.7	
CNN extracted feature	0.77 ± 0.45	0.67 ± 0.47	0.72 ± 0.45
**Fold 3**			
Age (year)		38.4 ± 11.5	
BMI (kg/m^2^)		24.4 ± 4.8	
Female		54.1	
CNN extracted feature	0.85 ± 0.37	0.62 ± 0.49	0.76 ± 0.43

**Table 5:** Association of the convolutional neural network’s (CNN) extracted features for various voice types and recent COVID-19 after controlling for age, BMI and sex.

	Sentence		Ah sound		Cough sound	
	Coefficient	*p* value	Coefficient	*p* value	Coefficient	*p* value
**Fold 1**						
Age (year)	0.07 ± 0.05	0.20	0.05 ± 0.05	0.31	0.05 ± 0.05	0.29
BMI (kg/m^2^)	0.01 ± 0.09	0.93	0.08 ± 0.08	0.31	0.06 ± 0.10	0.56
Female	− 2.77 ± 1.23	< 0.05	− 2.18 ± 1.00	< 0.05	− 2.64 ± 1.37	0.11
CNN extracted feature	3.47 ± 1.70	< 0.05	1.49 ± 0.80	0.06	5.00 ± 2.74	0.10
**Fold 2**						
Age (year)	0.08 ± 0.05	0.12	0.08 ± 0.05	0.14	0.11 ± 0.05	0.03
BMI (kg/m^2^)	− 0.03 ± 0.10	0.75	− 0.03 ± 0.09	0.74	0.01 ± 0.08	0.88
Female	− 1.69 ± 0.94	0.07	− 1.30 ± 0.91	0.16	− 1.66 ± 0.83	< 0.05
CNN extracted feature	2.24 ± 0.97	< 0.05	1.91 ± 0.86	< 0.05	− 0.41 ± 0.82	0.62
**Fold 3**						
Age (year)	0.19 ± 0.10	0.06	0.20 ± 0.10	< 0.05	0.19 ± 0.08	< 0.05
BMI (kg/m^2^)	− 0.04 ± 0.11	0.70	0.05 ± 0.10	0.64	0.01 ± 0.10	0.90
Female	− 4.17 ± 2.02	< 0.05	− 3.86 ± 2.05	0.06	− 3.18 ± 1.64	< 0.05
CNN extracted feature	2.29 ± 1.28	0.07	1.36 ± 1.12	0.22	0.45 ± 1.09	0.68

## Discussion

In the present study, we demonstrated that voice features represented by mel-spectrogram could distinguish patients with recent COVID-19 disease from controls, particularly with polysyllabic sentences. The results suggest that the SARS-CoV-2 may affect tissue involved in voice production well beyond the resolution of the disease. Some unique characteristics of COVID-19 such as loss of smell and taste^8^ have been described. However, to our knowledge, the alteration in voice has been less reported. It is also important to point out that such alteration is subclinical, not obvious to either the patients or healthcare providers. For the loss of smell and taste, early resolution was reported in most patients but the abnormality can persist in some patients up to 4 weeks after the onset of symptoms^9^. Our study showed that the subtle change in voice could be present even 60 days after being discharged from hospital. Recently, it has been increasingly aware that some symptoms of COVID-19 can persist well beyond the recovery in infected subjects. Long COVID was characterized by symptoms of fatigue, headache, dyspnea and anosmia and was more likely with increasing age and body mass index and female sex^10^ and is thought to occur in approximately 10% of people infected^11,12^. However, how soon and for how long the alteration can be detected is currently unknown. Further studies are warranted, particularly to evaluate the presence of voice change early in the course of the disease, which, if present and specific, could be developed into a screening modality for long COVID.

Our results are in keeping with previous studies suggesting that perturbation of voice has recently been suggested as a manifestation of COVID-19 which can occur in up to a quarter of patients with mild to moderate disease^13^. There are several factors which potentially can be responsible for the changes in voices in COVID-19. Besides being a consequence of inflammation of related voice producing organs, SAR-CoV2 can potentially affect the vocal cords directly. Enriched expression of ACE2, the receptor for SAR-CoV2 has been demonstrated in the head and neck regions, particularly in sinuses, salivary glands, oral cavity epithelial cells and vocal cords^14^. Moreover, post viral vagal neuropathy could occur which can present as persistent shortness of breath despite normal chest radiograph^15^.

Current artificial intelligence models can achieve diagnostic performance comparable to those of medical experts in various domains^16–18^. In the present study, we demonstrated that voice features such as mel-spectrogram can be represented as an image and used as inputs for CNN. For the classification of images, a number of feature visualizations have been explored to better understand how CNN sees features in images^19^. These learned features are usually hard to identify and interpret from a human vision perspective, causing a lack of understanding of the CNN’s internal working mechanism. Similarly, features in the mel-spectrum which distinguish individuals with past COVID-19 and controls in the present study are unclear. This ‘black box’ nature of deep neural networks is one of its shortcomings and the deep understanding of features contributing to classification performance is difficult to attain.

There have been many attempts to use voices as biomarkers for diseases including Parkinson’s disease^20^, heart failure^21^, and diabetes mellitus^22^. Currently there is no consensus on which kinds of speech or voice are more suitable for use as voice markers. For example, voice biomarkers for diabetes are varied in the literature and include matched fragments of speech^23^, free speech^24^ or vowel sounds^25^. The relative accuracy of using different kinds of human voices for such purposes are currently unclear. However, we demonstrated in the present study that speech utterances of a complex sentence are more accurate for the prediction of previous COVID-19 infection than simple vowels or a cough sound. The underlying basis for this difference is not clear, but it may be related to the higher variation in voice features from more complex sounds which render it more effective when used for classification by machine learning methods. To explore such a notion, we further analyzed the voice types according to their Shannon entropy. Originated from information theory, Shannon entropy is a measure to reflect information content of the variable under study^26,27^. For the proposed features selection methodology in machine learning, almost all the information-theoretic approaches are based on Shannon entropy^28^. Both the polysyllabic and the ‘ah’ sounds in the present study had higher Shannon entropy than the cough sound which corresponded with their apparent better performance than the cough sound. Moreover, as participants were instructed to produce sustained vowels with a continuous phonation over a certain time, it may introduce discontinuities in the pulmonic airstream in COVID-19 infected participants leading to sporadic, unintended interruptions of phonation when expressed the polysyllabic and the ‘ah’ sounds as compared to the cough sound^29^. Interestingly, as far as we know, most of the studies using voice to classify the presence of COVID-19 have utilized cough sounds as the study features^30–32^. It is therefore worthwhile to further explore speeches and other voice types which may have higher information content and better classification performance than cough sounds per se. Moreover, it is of note that regardless of different accuracies, all 3 voice types produced higher sensitivity compared to specificity, this would suggest that the practical use case of voices to classify past COVID would be more appropriate for screening purpose and caution should be exercised with negative results as false negative rates could be relatively high.

There are some limitations to the present study. First, the sample size was relatively small. However, we used transfer learning with a pre-trained model to mitigate this limitation. Second, baseline characteristics were not well matched across the two participant groups. However, after controlling for unmatched clinical parameters, the polysyllabic sentence used in this study was effectively used to distinguish patients with recent COVID-19 from controls. Third, there are a number of neural network architectures suggested for audio classification^33,34^, however only the VGG19 CNN was explored in this study. Future studies with a larger sample size, better-matched baseline characteristics between cases and controls, and varying neural network architecture are warranted.

## Conclusion

Deep learning is able to detect the subtle change in voice features of COVID-19 patients after recent resolution of the disease.

## Data Availability

The datasets generated and/or analysed during the present study are available from the corresponding author upon reasonable request.
